# Assessing the Impact of Health Literacy on Education Retention of Stroke Patients

**DOI:** 10.5888/pcd11.130259

**Published:** 2014-04-03

**Authors:** Kalina Sanders, Loretta Schnepel, Carmen Smotherman, William Livingood, Sunita Dodani, Nader Antonios, Katryne Lukens-Bull, Joyce Balls-Berry, Yvonne Johnson, Terri Miller, Wayne Hodges, Diane Falk, David Wood, Scott Silliman

**Affiliations:** Author Affiliations: Loretta Schnepel, Nader Antonios, Wayne Hodges, Diane Falk, Scott Silliman, University of Florida, College of Medicine, Gainesville, Florida; William Livingood, Sunita Dodani, David Wood, University of Florida, College of Medicine, Gainesville, Florida, and the Center for Health Equity and Quality Research, Jacksonville, Florida; Carmen Smotherman, Katryne Lukens-Bull, Joyce Balls-Berry, the Center for Health Equity and Quality Research, Jacksonville, Florida; Yvonne Johnson, Terri Miller, University of Florida Health Jacksonville, Jacksonville, Florida.

## Abstract

**Introduction:**

Inadequate health literacy is a pervasive problem with major implications for reduced health status and health disparities. Despite the role of focused education in both primary and secondary prevention of stroke, the effect of health literacy on stroke education retention has not been reported. We examined the relationship of health literacy to the retention of knowledge after recommended stroke education.

**Methods:**

This prospective cross-sectional study was conducted at an urban safety-net hospital. Study subjects were patients older than 18 admitted to the hospital stroke unit with a diagnosis of acute ischemic stroke who were able to provide informed consent to participate (N = 100). Health literacy levels were measured by using the short form of Test of Functional Health Literacy in Adults. Patient education was provided to patients at an inpatient stroke unit by using standardized protocols, in compliance with Joint Commission specifications. The education outcomes for poststroke care education, knowledge retention, was assessed for each subject. The effect of health literacy on the Stroke Patient Education Retention scores was assessed by using univariate and multivariate analyses.

**Results:**

Of the 100 participating patients, 59% had inadequate to marginal health literacy. Stroke patients who had marginal health literacy (mean score, 7.45; standard deviation [SD], 1.9) or adequate health literacy (mean score, 7.31; SD, 1.76) had statistically higher education outcome scores than those identified as having inadequate health literacy (mean score, 5.58; SD, 2.06). Results from multivariate analysis indicated that adequate health literacy was most predictive of education outcome retention.

**Conclusions:**

This study demonstrated a clear relationship between health literacy and stroke education outcomes. Studies are needed to better understand the relationship of health literacy to key educational outcomes for primary or secondary prevention of stroke and to refine stroke education for literacy levels of high-risk populations.

## Introduction

Inadequate health literacy is a pervasive problem with major implications for reduced health status and health disparities (1). However, the relationship between health literacy and incidence of stroke is not well understood. Stroke is the fourth leading cause of death in the United States and the leading cause of acquired physical disability in American adults (2,3). Approximately 795,000 cases of stroke are reported in the United States each year. Of these cases, 185,000 (23%) are recurrent strokes (2,3). Risk of stroke recurrence is influenced by modifiable factors, such as cigarette smoking, patient compliance with oral antihypertensive and antithrombotic medication, and use of statins (4). Many of these risk factors are influenced by the ability of patients to understand, initiate, and adhere to the instructions of their treating health care professionals.

Health literacy — the ability to obtain, process, and understand health information and services needed to promote better health (5) — may be a major barrier to the ability of stroke patients to reduce their risk of stroke. Patients with inadequate health literacy report less understanding of their medical conditions (6–8). Additionally, low literacy is associated with problems in using preventive services (9), delayed diagnosis (10), low self-management skills (11), lack of understanding medical instructions (12,13), and compliance with recommended treatments (14). Consequently, the US Surgeon General identified improvement of health literacy as a national priority (15).

Education is a fundamental strategy of stroke prevention and treatment, but the effect of health literacy on stroke education outcomes has not been reported. The purpose of this study was to assess the relationship of health literacy to stroke education outcomes, following stroke education in an acute stroke unit at an urban safety net hospital in Jacksonville, Florida. A secondary aim was to assess the relationship of demographic and other characteristics associated with levels of health literacy and to explore their relationship to education outcomes in ischemic stroke patients at the time of their hospital discharge.

## Methods

### Study design

This was a hospital-based, prospective, cross-sectional study. Health literacy levels were measured by using the short form of Test of Functional Health Literacy in Adults (S-TOFHLA) (16). Patient education was provided on the stroke unit at University of Florida Health–Jacksonville (UF Health–Jacksonville) by using standardized protocols, in compliance with Joint Commission specifications. The education sessions occurred as part of routine patient care. The subjects’ level of retention of poststroke care education was assessed on day of discharge by asking a series of 5 questions that were derived from the stroke performance measures on stroke education as required by the Joint Commission for certified stroke centers.

### Participants

From August 2009 through June 2010, 101 eligible patients with acute ischemic stroke (AIS) who were consecutively admitted to the inpatient stroke unit at UF Health–Jacksonville provided written consent to participate in the study. UF Health–Jacksonville is a 750-bed tertiary-care hospital located north of downtown Jacksonville, Florida, with an AIS catchment area that includes the city of Jacksonville, 10 northeast Florida rural counties, and 2 southeast Georgia rural counties. This hospital has been certified as a primary stroke center by the Joint Commission since 2004.

Study subjects were patients older than 18 who were admitted to the hospital stroke unit with an AIS diagnosis. Subjects were identified and recruited by either the treating neurologist or a stroke unit nurse manager. Diagnosis of AIS was confirmed by a neurologist, and a computerized tomography (CT) or magnetic resonance imaging (MRI) scan confirmed the location and size of the brain infarction. Subjects who were unable to provide informed consent were excluded. Prisoners and subjects with diagnosed severe cognitive dysfunction due to dementia, global aphasia, or receptive aphasia were excluded. Once subjects were identified and their consent was obtained, subjects underwent literacy assessments. Of 189 subjects screened, 70 were excluded due to severe cognitive impairment. Eighteen eligible subjects refused to participate, 1 subject withdrew consent before completing the assessment, and 100 (84%) completed the study that constituted our study sample.

Demographic information, including age, race/ethnicity, annual household income, and education level, was collected through either patient interview or chart review. Residences of patients were coded and analyzed using zip codes, which have been aggregated into the city of Jacksonville’s 6 health zones. These zones, designated by the county health department and that reflect different racial/ethnic and other demographic characteristics of the residents, were based on regional boundaries used by city organizational entities, including the Duval County Public Schools, Jacksonville Sheriff’s Office, and the Community Planning Action Councils, as well as physical geographic boundaries and barriers (river, ocean). Growing recognition of the effect of community of residence (place matters) on health outcomes, particularly in the Jacksonville community (17), makes this demographic factor an important confounding variable.

The magnitude of neurological deficit at the time of hospital admission for each subject was delineated by the National Institutes of Health Stroke Scale (NIHSS) score (18). When not documented, scores were assigned by one of the study neurologists via retrospective review of the admission physical examination. Additionally, time between stroke onset and discharge from hospital and hospital length of stay in days was noted. Each study subject was administered a Mini-Mental Status Exam (MMSE), a test of cognitive impairment, after obtaining consent (19).

### Literacy assessment

A trained research staff member administered the S-TOFHLA, which takes 8 minutes on average to administer (16). Briefly, it is a reliable, validated instrument used to assess a patient’s health literacy level (Cronbach’s α = 0.97; correlation with REALM [Rapid Estimate of Adult Literacy in Medicine]: *r* ^2^ = 0.81) (16). The S-TOFHLA contains 36 reading comprehension items. The health-related passages on the S-TOFHLA use a modified cloze procedure, where every fifth to seventh word is omitted and subjects select the correct word from among a set of 4 options. Each selection was scored a 1 for correct or a 0 for incorrect. Scores were summed to create a total score. The passages contain information about an upper gastrointestinal tract X-ray procedure and the “rights and responsibilities” section from a Medicaid application; the passages have Gunning-Fox readability indices of fourth and tenth grade, respectively. The 36-point scale of the S-TOFHLA is divided into 3 categories of functional literacy: inadequate (0–16), marginal (17–22), and adequate (23–36).

### Educational session

Nurses on the stroke unit are trained in stroke patient education via completion of a self-learning module with yearly skill updates. Upon admission to the stroke unit, the registered nurse (RN) assessed the patient’s readiness for learning and developed an individualized stroke education plan. Stroke education was initiated as soon as possible during patients’ stay in the stroke unit. Educational sessions were designed to cover all of the topics that the Joint Commission mandates for a certified primary stroke center: personal risk factors for stroke, warning signs for stroke, activation of emergency medical services, need for follow-up after discharge, and medications prescribed for stroke prevention (20). The RN educator provided both verbal and written stroke education by using standardized American Stroke Association–approved handouts as learning tools during the patient’s hospital stay. Patients usually received 30 to 60 minutes of individualized stroke education that included their personal risk factors and lifestyle modification.

### Retention assessment

After the education session and before discharge a research staff member assessed the patients’ comprehension and retention of the information provided in the educational session by asking the 5 questions of the Stroke Patient Education Retention (SPER) score (Table 1). All retention assessments occurred on day of discharge. The SPER scores range from 0 to 10. Questions 1 and 2 were scored 0 = no correct content in response to the question, 1 = the patient provided some but not all of the correct content in response to the question, and 2 = patient provided all key content in response to the question. Questions 3–5 responses were scored 0 = no correct content in response to the question, and 2 = the patient provided the correct response to the question. A score of 10 indicates all correct responses for key content in the education sessions.

**Table 1:** Stroke Patient Education Retention (SPER) Items and Responses^a^

Question	Acceptable Responses
1. Do you know the warning signs for stroke?	Sudden weakness/numbness one side of the body
	Confusion/trouble speaking or understanding
	Trouble seeing
	Trouble walking/dizziness or loss of balance
	Severe headache

2. What are your personal risk factors for stroke that can be modified to lower your risk of another stroke?	Hypertension
	Diabetes mellitus
	High cholesterol
	Tobacco use
	Carotid artery disease
	Atrial fibrillation

3. What will you do if you develop symptoms of a stroke?	Seek emergency services by calling 911

4. Do you know the medication you were prescribed to prevent another stroke?	Name of antiplatelet drug or anticoagulation drug

5. What type of stroke did you have?	Ischemic stroke
	Brain infarction
	Stroke due to an occluded blood vessel

a Subject responses were scored (range, 0–10) using the Stroke Patient Education Retention (SPER) score. Questions 1 and 2 were scored as follows: 0 = no correct content in response to the question, 1 = the patient provided some but not all of the correct content in response to the question, and 2 = patient provided all key content in response to the question. Questions 3–6 were scored as follows as 0 = no correct content in response to the question and 2 = the patient provided the correct response to the question. A score of 10 indicates all correct responses to key content in the education sessions.

### Data analysis

Statistical analyses and modeling were conducted using SAS version 9.3 software (SAS Institute, Inc, Cary, North Carolina). Continuous variables were described by using means, standard deviations (SDs), and medians. Categorical variables were presented as counts and percentages and analyzed using Pearson χ^2^ or Fisher Exact tests, when appropriate. Univariate and multivariable analyses were used to identify factors independently associated with SPER. For univariate analysis, the differences in the SPER scores among the 3 S-TOFHLA groups were assessed using the nonparametric Kruskal–Wallis test and with Tukey’s adjustment for pairwise comparisons. For multivariate analysis, analysis of covariance (ANCOVA) was used to model the data and estimate the least squared means. A backward elimination variable procedure was conducted to eliminate weak predictors that contributed marginally in explaining variation in the SPER scores. Variables considered included S-TOFHLA score, sex, age (<60 years or ≥60 years), income (<$25,000 per year or ≥$25,000 per year), education (less than high school graduate or high school graduate or more), race (African American or other than African American), employment status (currently working, retired, unemployed, or other), NIHSS score, and MMSE score.

## Results

### Subjects

Variations in health literacy across the various patient demographic characteristics were not significant except for education level (*P* = .02) and MMSE score (*P* = .003) (Table 2). The mean education SPER score was 6.7 (SD, 2.0). Subjects with adequate health literacy outperformed the other 2 categories of health literacy on all 5 cognitive retention items (Table 3). Subjects who provided all key content in response to each of the SPER questions with marginal or adequate health literacy were compared with those with inadequate health literacy. Differences between having adequate health literacy and inadequate health literacy and between having marginal health literacy and inadequate literacy on the SPER mean scores were significant, but differences between adequate and marginal health literacy were not significant (Table 4).

**Table 2:** Patient Characteristics Related to Health Literacy Levels,^a^ Jacksonville, Florida, August 2009–June 2010

Characteristic	Total^b^	S-TOFHLA Levels			*P* Value
		Inadequate	Marginal	Adequate	
**Total**	100 (100)	35 (37)	21 (22)	39 (41)	NA
**Sex**					
Male	57 (57)	20 (57)	9 (43)	25 (64)	.29^c^
Female	43 (43)	15 (43)	12 (57)	14 (36)	
**Age, y**					
≥60	48 (48)	20 (57)	10 (48)	14 (36)	.19^c^
<60	52 (52)	15 (43)	11 (52)	25 (64)	
**Income, $**					
<25,000/y	64 (75)	24 (77)	10 (59)	28 (80)	.23^c^
≥25,000/y	21 (25)	7 (23)	7 (41)	7 (20)	
**Education**					
Less than high school graduate	56 (57)	25 (74)	12 (57)	16 (41)	.02^c^
High school graduate or more	43 (43)	9 (26)	9 (43)	23 (59)	
**Race**					
African American	56 (56)	20 (57)	12 (57)	21 (54)	.95^c^
Other than African American	44 (44)	15 (43)	9 (43)	18 (46)	
**NIHSS score**					
≤3	60 (64)	20 (59)	13 (65)	25 (71)	.55^c^
>4	34 (36)	14 (41)	7 (35)	10 (29)	
**Employment status**					
Currently working	23 (23)	6 (17)	4 (19)	12 (31)	.28^d^
Retired	36 (36)	17 (49)	7 (33)	9 (23)	
Unemployed	27 (27)	7 (20)	6 (29)	14 (36)	
Other	14 (14)	5 (14)	4 (19)	4 (10)	
**Duval County Health Zone**					
1 = Urban core	43 (43)	19 (54)	8 (38)	14 (36)	.16^d^
2 = Greater Arlington	5 (5)	1 (3)	1 (5)	3 (8)	
3 = Southeast	5 (5)	0	2 (10)	3 (8)	
4 = Southwest	13 (13)	2 (6)	4 (19)	5 (13)	
5 = Outer rim	8 (8)	6 (17)	0 (0)	2 (5)	
6 = Beaches	0	0	0	0	
7 = Out of Duval	26 (26)	7 (20)	6 (29)	12 (31)	
**MMSE score, mean (SD)^e^**	26.4 (3.3)	24.9 (3.6)	26.8 (3.8)	27.6 (2.4)	.003^f^
**NIHSS score, mean (SD)^g^**	3.4 (2.9)	3.9 (3.1)	3.1 (2.4)	2.9 (3.1)	.28^f^

Abbreviations: S-TOFHLA, short form of Test of Functional Health Literacy in Adults; NA, not applicable; NIHSS, National Institutes of Health Stroke Scale; MMSE, Mini-Mental Status Exam; SD, standard deviation.

a Health literacy assessed by S-TOFHLA; values expressed as no. (%) unless otherwise indicated.

b Total counts may be higher than the total counts over S-TOFHLA groups because of 5 missing values for S-TOFHLA; total values for some categories may not calculate to 100 because of missing responses.

c Calculated by using Pearson χ^2^ test.

d Calculated by using Fisher Exact test.

e Calculated on the basis of responses to the MMSE; raw score range, 0–30.

f Calculated by using Kruskal–Wallis test.

g Calculated on the basis of clinical exam; raw score range, 0–42.

**Table 3:** Description of Stroke Patient Education (SPER) Outcome Scores,^a^ Jacksonville, Florida, August 2009–June 2010

Responses^b^	S-TOFHLA Group			Total
	Inadequate (n = 33)	Marginal (n = 20)	Adequate (n = 39)	
**Do you know the warning signs for stroke?**				
None	1 (3)	0	0	1 (1)
Some	29 (88)	18 (90)	32 (82)	84 (87)
All	3 (9)	2 (10)	7 (18)	12 (12)
**What are your personal risk factors for stroke that can be modified to lower your risk of another stroke?**				
None	9 (27)	0	3 (8)	12 (12)
Some	13 (39)	9 (45)	17 (44)	43 (45)
All	11 (33)	11 (55)	19 (49)	42 (43)
**What will you do if you develop symptoms of a stroke?**				
None	8 (24)	2 (10)	5 (13)	15 (15)
All	25 (76)	18 (90)	34 (87)	82 (85)
**Do you know the medication you were prescribed to prevent another stroke?**				
None	12 (36)	1 (5)	9 (23)	23 (24)
All	21 (64)	19 (95)	30 (77)	74 (76)
**What type of stroke did you have?**				
None	22 (67)	9 (45)	11 (28)	46 (47)
All	11 (33)	11 (55)	28 (72)	51 (53)
**Stroke education score, mean (SD)**	5.58 (2.06)	7.45 (1.61)	7.31 (1.76)	6.69 (2.0)

Abbreviations: S-TOFHLA, short form of Test of Functional Health Literacy in Adults; SD, standard deviation.

a Values expressed as no. (%) unless otherwise indicated.

b None = no correct content provided in response to the question; some = the patient provided some but not all of the correct content in response to the question; all = patient provided all key content in response to the question.

**Table 4:** Differences in Stroke Patient Education Retention Scores Between S-TOFHLA Levels, Jacksonville, Florida, August 2009–June 2010

Comparison of S-TOFHLA levels	Differences Between Mean Scores (95% CI)	*P* Value^a^
Marginal health literacy with inadequate health literacy	1.87 (0.63 to 3.12)	.001
Adequate health literacy with inadequate health literacy	1.73 (0.69 to 2.77)	.001
Marginal health literacy with adequate health literacy	0.14 (−1.07 to 1.35)	.60

Abbreviation: S-TOFHLA, short form of Test of Functional Health Literacy in Adults; CI, confidence interval.

a Tukey-adjusted *P* values.

Using multivariate analysis to examine the relationship of health literacy to education outcomes while considering other patient characteristics, a backward selection procedure was conducted to eliminate weak predictors that contributed marginally in explaining variation in the SPER scores using an ANCOVA regression modeling approach. The full model had STOFHLA score (*P* = .02), sex (*P* = .03), age (*P* = .95), income (*P* = .11), education (*P* = .43), race (*P* = .84), employment (*P* = .54), NIHSS score (*P* = .87), and MMSE score (*P* = .22) as predictors and the variables with the lower contribution (higher *P* value) to the model were excluded one by one. The significant predictors identified by the model were STOFHLA (*P* = .004), sex (*P* = .03), income (*P* = .007), and MMSE score (.04). The model explained 35% of the variation in SPER scores. Adjusting for all the other variables in the model and for multiple comparisons, subjects with marginal health literacy (adjusted mean SPER score = 7.59) and those with adequate health literacy (adjusted mean SPER score = 7.50) had better recall than subjects with inadequate health literacy (adjusted mean SPER score = 6.04) (*P* = .02 and *P* = .006 respectively). There was no significant difference estimated between subjects with adequate health literacy and subjects with marginal health literacy (*P* = .98) (Table 3).

## Discussion

Patients hospitalized with AIS are at significant risk for recurrent stroke over short- and long-term periods. A meta-analysis of hospital- and community-based studies reporting risk of stroke recurrence after first ever stroke found overall cumulative risk of 3.1% at 30 days, 11% at 1 year, 26% at 5 years, and 39% at 10 years (21). Barriers to good control of risk factors and medication compliance include inadequate follow-up and monitoring of stroke survivors by health care professionals, ineffective education of patients regarding risk modification and event prevention, and inadequate self-management by patients (22), all of which can be influenced by health literacy and knowledge retention, the focus of this study. We found that subjects with AIS and inadequate health literacy had a significantly lower stroke education retention rate than AIS subjects with marginal or adequate literacy. Those with inadequate health literacy, on average, recalled only half of the stroke educational material provided to them. Additionally, 9 of the 12 responders who could not name any of their personal risk factors had inadequate health literacy. These findings document the potential adverse effect of low health literacy on patient adherence to self-management in the subgroup of AIS patients.

Completion of stroke education on AIS inpatients before hospital discharge is 1 of the 8 core performance measures that Joint Commission–certified primary stroke centers must conduct. There are more than 1,000 certified primary stroke centers in all 50 states (23). Many of these certified stroke centers are urban safety-net hospitals, such as UF Health–Jacksonville, and provide a disproportionately higher amount of care to patients with lower income and lower educational levels. Even after receiving recommended education, only 12% of the subjects in our study could identify all 5 warning signs for a stroke, 43% could name all of their personal risk factors, and fewer than half could identify the type of stroke they suffered. These findings have implications for stroke education being used at urban stroke centers.

Future research is needed on education approaches that are modified to accommodate differences in literacy and culture. From our reviews, no other studies have reported the influence of health literacy level on education retention in hospital inpatients with AIS. Community-based and single-center studies have suggested that lower heath literacy is associated with less knowledge regarding primary prevention of stroke. Significant deficiencies in stroke literacy, including awareness of stroke warning symptoms and risk factors, in a population at risk for low health literacy have also been described (24,25). Patients with atrial fibrillation and limited health literacy who attended an anticoagulation clinic were also less likely to describe a stroke warning sign and to know that their condition increased their risk for stroke than those attendees with adequate health literacy (26). Thus far, no study has evaluated the effect of adjusting primary stroke prevention or poststroke education for lower levels of health literacy on knowledge retention.

In addition to the inverse relationship between low literacy and positive education outcomes, our study showed that low health literacy was prevalent in this high-risk population. We found that 59% of the population had marginal to inadequate health literacy at the time of discharge. This percentage of patients with low health literacy was much greater than would be expected in a general population mix. A previous population-based study using S-TOFHLA reported that 30% of the non-Hispanic English-speaking population scored in the marginal to inadequate range (27). We found a significant correlation between health literacy levels with age and MMSE, 2 factors that would be expected to be more correlated with literacy. Our study was consistent with previous studies looking at the relationship between S-TOFHLA and the MMSE (28). Results of our study reinforce those of other studies that indicate that literacy is a factor contributing to health disparities, both in terms of prevalence of low literacy and the impact of low literacy on education outcomes.

A limitation of our study was the lack of evidence or predictive validity concerning stroke patient education outcomes, such as long-term retention and patient adherence or fidelity to self-management. Although researchers have found associations between education-related outcomes such as understanding and behavior, the relationship between improved education outcomes and improved adherence to stroke prevention strategies and reduced stroke recurrence has not been well established. More study may be needed to clarify what patient education outcomes are most effective in increasing adherence and improving patient self-management. Another limitation of this study was that the modeling approach was not sufficiently powered (sample size not large enough) to detect the effect of many demographic factors on stroke incidence. Consequently, only the most prominent factors that influenced patient education retention were statistically significant.

Literacy is but one factor that can contribute to patient adherence to follow-up care to prevent and control for the recurrence of stroke and to achieve optimal functioning following a stroke incident. As with most human behavior, adherence is likely to be influenced by the complex and interacting relationships of social, physical, intellectual, and emotional factors. Developing the evidence base for achieving better stroke outcomes requires better understanding of the factors that influence adherence to recommended patient behaviors, and health literacy has been demonstrated to be a factor that influences patient ability to retain clinical education and counseling. Developing approaches to education and counseling that adjust to the individual circumstance of each patient are likely to be needed, given the variation in patient profiles. Educational and counseling interventions that account for differences in literacy may be necessary in the same way that cultural tailoring (29,30) may be important to improve outcomes and reduce disparities. Focusing research on education of caretakers and tailoring the education interventions to the literacy and culture of the caretakers are other areas of research that are likely to be needed to improve stroke outcomes. It is clear that active adherence of patients and their caretakers is needed to achieve better outcomes, and literacy cannot be ignored in the quest for stroke prevention and control of stroke-related risk factors. Given the overlap of education outcomes for stroke treatment with population health education for stroke, the need to study and adjust stroke education for different levels of literacy, particularly for high-risk populations and populations with health disparities, is warranted.
